# Performance of the Models Predicting In-Hospital Mortality in Patients with ST-Segment Elevation Myocardial Infarction with Predictors in Categorical and Continuous Forms

**DOI:** 10.17691/stm2024.16.1.02

**Published:** 2024-02-28

**Authors:** K.I. Shakhgeldyan, N.S. Kuksin, I.G. Domzhalov, B.I. Geltser

**Affiliations:** Associate Professor, Director of the Institute of Information Technologies; Vladivostok State University, 41 Gogolya St., Vladivostok, 690014, Russia; Head of the Laboratory of Big Data Analysis in Medicine and Healthcare; Far East Federal University, 10 Ayaks Village, Russkiy Island, Vladivostok, 690922, Russia; PhD Student, Institute of Mathematics and Computer Technologies; Far East Federal University, 10 Ayaks Village, Russkiy Island, Vladivostok, 690922, Russia; PhD Student, School of Medicine and Life Sciences; Far East Federal University, 10 Ayaks Village, Russkiy Island, Vladivostok, 690922, Russia; Professor, Corresponding Member of the Russian Academy of Sciences, Deputy Director of School of Medicine and Life Sciences; Far East Federal University, 10 Ayaks Village, Russkiy Island, Vladivostok, 690922, Russia

**Keywords:** prognostic models, data categorization, ST-segment elevation myocardial infarction, mortality, risk factors, method of Shapley additive explanation

## Abstract

**The aim of the study** is to assess the performance of predictive models developed on the basis of predictors in the continuous and categorical forms to predict the probability of in-hospital mortality (IHM) in patients with ST-segment elevation myocardial infarction (STEMI) after percutaneous coronary intervention (PCI).

**Materials and Methods:**

A single-center retrospective study has been conducted, within the framework of which data from 4674 medical records of patients with STEMI after PCI, treated at the Regional Vascular Center of Vladivostok (Russia), have been analyzed. Two groups of patients were identified: group 1 consisted of 318 (6.8%) individuals who died in the hospital, group 2 included 4356 (93.2%) patients with a favorable outcome of treatment. IHM prognostic models were developed using multivariate logistic regression (MLR), random forest (RF), and stochastic gradient boosting (SGB). 6-metric qualities were used to evaluate the accuracy of the models. Threshold values of IHM predictors were determined using a grid search to find the optimal cut-off points, calculating centroids, and Shapley additive explanations. The latter helped evaluate the degree to which the model predictors influence the endpoint.

**Results:**

Based on the results of the multi-stage analysis of indicators of clinical and functional status of the STEMI patients, new predictors of IHM have been identified and validated, complementing the factors of the GRACE scoring system, their categorization has been carried out and prognostic models with continuous and categorical variables based on MLR, RF, and SGB have been developed. These models had a high (AUC — 0.88 to 0.90) and comparable predictive accuracy, but their predictors differed in various degrees of influence on the endpoint. The comparative analysis has shown that the Shapley additive explanation method has advantages in categorizing predictors compared to other methods and allows for detailing the structure of their relationships with IHM.

**Conclusion:**

The use of modern data mining methods, including machine learning algorithms, categorization of predictors, and assessment of the degree of their effect on the endpoint, makes it possible to develop predictive models possessing high accuracy and the properties of explanation of the generated conclusions.

## Introduction

Today, one of the effective methods of treating ST-segment elevation myocardial infarction (STEMI) is revascularization of myocardium using percutaneous coronary intervention [1]. However, in-hospital mortality (IHM) rate after emergency percutaneous coronary intervention (PCI) remains high from 4 to 7% making the need for predicting unfavorable events crucial [2].

The GRACE scoring system (Global Registry of Acute Coronary Events) is referred to the most required tools of riskometry, the improvement of which is the aim of a number of investigations of the last years [3–6]. In the majority of cases, the base factors of this system are complemented with new predictors to build prognostic models, and the correct selection of their threshold values remains important.

At present, methods of predictive analytics based on machine learning, which are increasingly being applied in different field of medicine, may be used to solve this task [7–10]. At the same time, implementation of the machine learning models into clinical practice is rather limited due to their “nontransparency”. This problem may be solved by means of categorization of continuous variables used in prognostic algorithms. Data categorization makes it possible to determine threshold values of the analyzed indicators, the deviations in which may be used for detection of the risk factors and clinical reasoning of predicted probability of unfavorable events [11, 12]. Besides, by combining the risk factors one can realize the possibility of characterizing a complex impact of various features on the response variable [13]. Moreover, some authors believe that indicators in the categorical form, dichotomized in particular, may lead to the loss of information, distortion of the analysis results [14–16], increased share of false-positive [17] and false-negative [18] conclusions. Despite the indicated drawbacks, recommendations of the STROBE (Strengthening the reporting of observational studies in epidemiology) confirm the appropriateness of using data categorization provided that the methods of its implementation are indicated [19].

**The aim of the study** is to assess the performance of predictive models built on the basis of predictors in the continuous and categorical forms to predict the probability of in-hospital mortality in patients with ST-segment elevation myocardial infarction after percutaneous coronary intervention.

## Materials and Methods

### Characteristics of patients

A single-center retrospective cohort study has been carried out, within the scope of which medical records of 4674 patients (3200 men and 1474 women) with STEMI at the age of 26–93 years (median — 63 years, 95% confidence interval (CI): 62–63), have been analyzed. Patients were treated in the Regional Vascular Center “Primorskiy Territory Clinical Hospital No.1” (Vladivostok, Russia) in the period from 2015 to 2021 [20]. All patients underwent emergency PCI. Patients were divided into two groups: group 1 comprised 318 patients (6.8%) died in the hospital; group 2 included 4356 patients (93.2%) with a favorable outcome. The study was conducted in compliance with the Declaration of Helsinki and approved by the local ethical committee of the Far Easten Federal University (Vladivostok, Russia), Protocol No.8 of June 8, 2023.

Patients with validated STEMI and PCI performed on the first day of the inpatient treatment met the criteria of inclusion into the study. Exclusion criteria were as follows: unstable angina, non-ST-elevation myocardial infarction, and absence of indications for PCI.

Clinical and functional status of the patients was evaluated on the first day of hospital treatment using 136 factors, the main of which are presented in Table 1. The data included 5 features from the GRACE scoring system: patients’ age, acute heart failure (AHF) according to Killip classification, heart rate (HR), systolic blood pressure (SBP), creatinine concentration in blood serum. Indicators of the laboratory tests have been also analyzed: content of erythrocytes (RBC), leukocytes (WBC), lymphocytes (LYM), neutrophils (NEUT), eosinophils (EOS); hemoglobin (Hb), thrombocytes (PLT), and thrombocrit (PCT); international normalized ratio (INR); thrombin time (TT); prothrombin index (PTI), activated partial thromboplastin time (APTT); levels of fibrinogen and glucose in blood serum. Postoperative echocardiographic examination included determination of the longitudinal and transverse dimensions of left and right atrium (LA1, LA2 and RA1, RA2), end systolic (ESD) and diastolic (EDD) dimension of the left ventricle (LV), left ventricular ejection fraction (LV EF) using the Teichholz formula, mean pulmonary artery pressure (mPAP). The following estimate indicators have been assessed: relative myocardium mass index of the left ventricle (LV RMMI), relative posterior wall thickness (RPWT) of the LV, body mass index (BMI).

**Table 1:** Clinical and functional description of patients with ST-segment elevation myocardial infarction

Predictor	Group 1 (n=318)	Group 2 (n=4356)	OR (95% CI)	p
Females, n (%)	142 (44.65)	1332 (30.58)	1.8 (1.5–2.3)	<0.000001
Age (years), Me [Q1; Q3]	71 [63; 78]	62 [55; 69]	—	<0.000001
Height (cm), Me [Q1; Q3]	168 [164; 174]	170 [165; 176]	—	0.000001
Body mass (kg), Me [Q1; Q3]	78 [70; 85]	80 [71; 90]	—	0.000012
BMI, Me [Q1; Q3]	27.04 [26.10; 27.68]	27.68 [27.46; 27.70]	—	0.082
SBP (mm Hg), Me [Q1; Q3]	110 [90; 130]	130 [120; 150]	—	<0.000001
DBP (mm Hg), Me [Q1; Q3]	70 [60; 80]	80 [75; 90]	—	<0.000001
PBP (mm Hg), Me [Q1; Q3]	40 [40; 42]	50 [50; 50]	—	<0.000001
HR per minute, Me [Q1; Q3]	86 [72; 100]	72 [65; 80]	—	<0.000001
Creatinine (μmol/L), Me [Q1; Q3]	130.0 [96.0; 193.3]	97.0 [81.0; 114.8]	—	<0.000001
AHF Killip class, n (%):				
without AHF	0	15 (0.34)	—	0.59
I	71 (22.33)	2726 (62.58)	0.17 (0.13–0.23)	<0.000001
II	58 (18.2)	867 (19.9)	0.90 (0.67–1.20)	0.50
III	66 (20.75)	479 (11.0)	2.1 (1.6–2.8)	<0.000001
IV	123 (38.7)	269 (6.18)	9.6 (7.4–12.4)	<0.000001
III–IV	189 (59.4)	748 (17.2)	7.1 (5.6–9.0)	<0.000001
LV EF (%), Me [Q1; Q3]	46.5 [38.0; 54.8]	56.0 [50.0; 61.0]	—	<0.000001
LV EDD (cm), Me [Q1; Q3]	5.0 [4.6; 5.5]	5.0 [4.7; 5.3]	—	0.35
LV ESD (cm), Me [Q1; Q3]	3.7 [3.2; 4.0]	3.4 [3.1; 3.8]	—	<0.000001
RPWT, Me [Q1; Q3]	0.408 [0.340; 0.470]	0.417 [0.380; 0.470]	—	0.554
LV RMMI, Me [Q1; Q3]	1.06 [0.84; 1.28]	0.96 [0.80; 1.14]	—	0.0003
mPAP (mm Hg), Me [Q1; Q3]	35.0 [28.25; 46.0]	28.0 [25.0; 30.0]	—	<0.000001
LA1 (cm), Me [Q1; Q3]	4.10 [3.80; 4.50]	3.9 [3.6; 4.2]	—	<0.000001
LA2 (cm), Me [Q1; Q3]	5.2 [4.8; 5.7]	4.9 [4.6; 5.2]	—	<0.000001
RA1 (cm), Me [Q1; Q3]	3.8 [3.5; 4.2]	3.6 [3.4; 3.9]	—	<0.000001
RA2 (cm), Me [Q1; Q3]	4.8 [4.5; 5.3]	4.7 [4.4; 5.0]	—	0.00004
WBC (10^9^/L), Me [Q1; Q3]	14.5 [10.9; 19.2]	9.8 [7.9; 12.3]	—	<0.000001
NEUT (%), Me [Q1; Q3]	81.30 [75.75; 86.50]	66.70 [59.10; 74.90]	—	<0.0001
LYM (%), Me [Q1; Q3]	10.7 [7.7; 15.9]	19.6 [13.5; 27.0]	—	<0.000001
EOS (%), Me [Q1; Q3]	0.1 [0; 0.3]	0.9 [0.3; 1.9]	—	<0.000001
RBC (10^12^/L), Me [Q1; Q3]	4.2 [3.8; 4.6]	4.5 [4.1; 4.8]	—	<0.000001
Hb (g/L), Me [Q1; Q3]	130 [114; 142]	141 [128; 152]	—	<0.000001
PLT (10^9^/L), Me [Q1; Q3]	228 [187; 288]	221 [185; 266]	—	0.02
Glucose (mmol/L), Me [Q1; Q3]	7.90 [6.30; 10.31]	5.8 [5.1; 7.0]	—	<0.000001
Urea (μmol/L), Me [Q1; Q3]	12.12 [8.70; 17.30]	6.70 [5.24; 8.84]	—	<0.000001
PCT (%), Me [Q1; Q3]	0.22 [0.17; 0.28]	0.20 [0.16; 0.24]	—	0.0012
PTI (%), Me [Q1; Q3]	75.5 [57.6; 87.0]	89.3 [79.7; 97.0]	—	<0.000001
INR (units), Me [Q1; Q3]	1.26 [1.10; 1.65]	1.06 [1.0; 1.16]	—	<0.000001
TT (s), Me [Q1; Q3]	21.9 [19.9; 30.4]	21.4 [19.5; 25.7]	—	0.012
APTT (s), Me [Q1; Q3]	39.7 [32.7; 58.2]	36.5 [32.2; 42.7]	—	0.000026
Anterior myocardial infarction, n (%)	177 (55.66)	2017 (46.30)	1.50 (1.16–1.83)	0.001
AF, n (%)	129 (40.57)	772 (17.72)	3.20 (2.51–4.02)	<0.000001
DM2, n (%)	100 (31.45)	830 (19.05)	1.90 (1.50–2.46)	<0.000001
CKD, n (%)	83 (26.1)	677 (15.5)	1.97 (1.50–2.60)	<0.000001
COPD, n (%)	25 (7.9)	354 (8.1)	0.96 (0.63–1.47)	0.95

The final point of the investigation was presented by the IHM indicator in STEMI patients after PCI from all causes in the form of categorical binary feature (“presence” or “development”).

### Methods of statistical analysis and machine learning

According to the Kolmogorov–Smirnov test, the data were not distributed normally, therefore, nonparametric statistical methods were applied. The indicators were presented as a median (Me) and interquartile intervals [Q1; Q3]; the Mann–Whitney test was used for intergroup comparison of continuous variables, while χ^2^ was applied for categorical ones. Odds ratio (OR) and their 95% CI was calculated using the Fisher’s exact test. Differences were considered statistically significant at p<0.01.

Models were developed using methods of multivariate logistic regression (MLR), random forest (RF), and stochastic gradient boosting (SGB). Their quality was estimated by six metrics: area under ROC-curve (AUC), sensitivity (Sen), specificity (Spec), F1-score, positive predictive value (PPV), negative predictive value (NPV).

To select the threshold values of potential predictors, methods of optimization on the grid with a pitch of Δ=(max–min)/100 were employed: minimization of p-value, Min(p), maximization of OR, Max(OR), and AUC, Max(AUC), method of centroids, and Shapley additive explanations (SHAP) [21, 22]. The latter was also used to evaluate the degree to which the model predictors influence the endpoint.

The dataset was divided into 2 samples: for training and cross-validation (80%), and for final testing (20%). The procedure of training and cross-validation was performed by stratification in k-Folders on 10 samples. The averaged quality metrics AUC, Sen, and Spec were used to choose the best model, select and validate predictors and select optimal hyper-parameters by grid search over acceptable values. For final testing, the best models of MLR, RF, and SGB with optimal parameters and hyper-parameters were trained on 80% of the dataset, and validated on the subgroup for final testing. To estimate the quality metrics by confidence regions, the procedure was repeated 500 times performing randomly the initial sampling using Monte Carlo method. The data analysis and model building were done in Python 3.9.16 with an open source code.

***Study design*** included 4 stages. At the first stage, a pool of potential IHM predictors was formed using the tests of intergroup comparisons (see Table 1).

At the second stage, methods of machine learning were used to develop prognostic IHM models with predictors in the continuous form including 5 basic factors of the GRACE scale. To improve the prognostic accuracy, the model structure was supplemented in a step-wise manner with new predictors selected at the first stage of the investigation provided that statistically significant difference was at the level p<0.01. The prognostic significance of the predictor was considered validated, if the AUC value increased after its inclusion into the model. At this stage, the degree of influence of the best model predictors on the study endpoint was analyzed using the SHAP method.

At the third stage, continuous predictors were categorized by means of different techniques in order to find the threshold values, the deviations from which would allow us to refer them to the IHM risk factors.

At the fourth stage, prognostic IHM models were generated based on the categorical predictors and evaluated in terms of their impact on the endpoint.

## Results

***At the first stage***, an intergroup analysis of clinical, demographic, and laboratory indicators has been carried out. The majority of them, including all predictors of the GRACE scale, had statistically significant differences (see Table 1). Elderly females short in height predominated among the dead patients. Besides, it was typical for patients of group 1 to have AHF Killip class III and IV; lower values of SBP, DBP, LV EF, LYM, EOS, PTI, RBC, Hb; higher levels of HR, mPAP, LV ESD, creatinine, NEUT, APTT, INR, urea, glucose; increased atrial dimensions and LV RMMI indicator. They more often suffered from anterior myocardial infarction, type 2 diabetes (DM2), atrial fibrillation (AF), and chronic kidney disease (CKD).

***At the second stage***, prognostic IHM models were generated, where new factors were used in the continuous form additionally to the 5 basis indicators of the GRACE scoring system. These factors were selected during iterative testing of the pool of potential predictors obtained at the first stage. Testing was performed by including alternately each potential predictor into the base model of the GRACE scale and leaving in the model the only one that gave maximal increase of the AUC metrics. In the next iterations, the procedure was repeated for the remaining potential predictors. In this way, 5 new prognostic factors were selected: LV EF, NEUT, EOS, PCT, and glucose. It should be also noted that the comparison of AUC values for the model previously built by us [23] using the same patient sample and the predictors of GRACE only and the model supplemented by new predictors, demonstrated a higher accuracy of the latter (AUC — 0.836 vs. 0.90).

The comparison of the predictive value of the models generated by MLR, SGB, and RF has shown that they possess a high prognostic capacity and have close values of quality metrics during cross-validation and final testing (AUC varied from 0.884 to 0.90). This shows the absence of their re-training and good generalization properties (Table 2).

**Table 2:** Assessment of accuracy of in-hospital mortality prognostic models for patients with ST-segment elevation myocardial infarction after percutaneous coronary intervention using predictors in the continuous form, Me [Q1; Q3]

Metrics	Cross-validation			Final testing		
	MLR	SGB	RF	MLR	SGB	RF
AUC	0.900 [0.885; 0.916]	0.891 [0.871; 0.911]	0.885 [0.870; 0.900]	0.900 [0.841; 0.959]	0.892 [0.834; 0.951]	0.884 [0.824; 0.944]
Sen	0.843 [0.810; 0.877]	0.825 [0.779; 0.872]	0.796 [0.749; 0.843]	0.843 [0.715; 0.972]	0.824 [0.692; 0.957]	0.798 [0.672; 0.925]
Spec	0.836 [0.824; 0.849]	0.816 [0.797; 0.835]	0.806 [0.784; 0.828]	0.838 [0.807; 0.868]	0.819 [0.783; 0.855]	0.806 [0.766; 0.846]
PPV	0.165 [0.152; 0.178]	0.115 [0.131; 0.161]	0.136 [0.124; 0.148]	0.168 [0.141; 0.194]	0.150 [0.125; 0.175]	0.138 [0.101; 0.167]
NPV	0.993 [0.992; 0.995]	0.992 [0.990; 0.994]	0.991 [0.989; 0.993]	0.993 [0.987; 0.999]	0.992 [0.986; 0.998]	0.991 [0.985; 0.996]
F1-score	0.275 [0.256; 0.295]	0.248 [0.226; 0.270]	0.231 [0.214; 0.249]	0.278 [0.239; 0.317]	0.253 [0.215; 0.291]	0.235 [0.189; 0.280]

**Figure 1.**
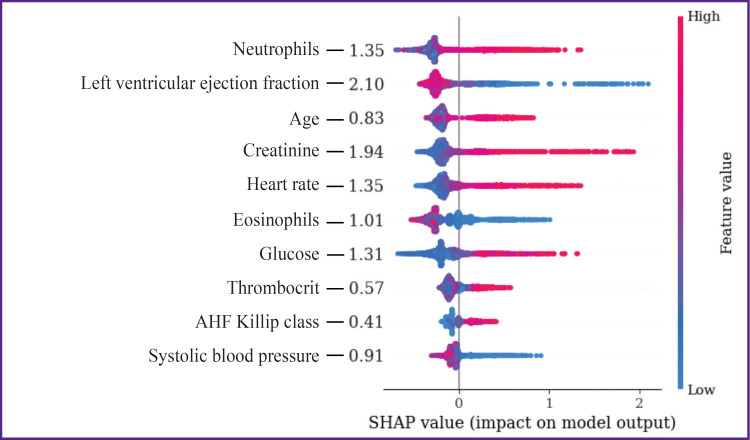
Assessment of importance of in-hospital mortality predictors in the continuous form in the model of multivariate logistic regression

Assessing the impact of continuous predictors on the endpoint using the SHAP method has shown that LV EF and creatinine have the closest association with IHM. HR, NEUT, and glucose influenced this point to a lesser degree, while indicators such as EOS, SBP, patients’ age, PCT, and AHF Kilipp class had the smallest effect on the endpoint (Figure 1).

***At the third stage of the study***, IHM predictors were categorized in the continuous form using grid search to find the optimal cut-off threshold, SHAP method, and centroid calculation. Application of the threshold values, the deviation from which is associated with the higher probability of IHM, allows for considering the categorized data as risk factors for adverse events. The risk factor is encoded as “1” with the postfix “+” if the predictor value exceeds the threshold, or with the postfix “–” if the value does not reach the threshold; in other cases, when the predictor value is in another range, the risk factor is encoded as “0”.

**Figure 2.**
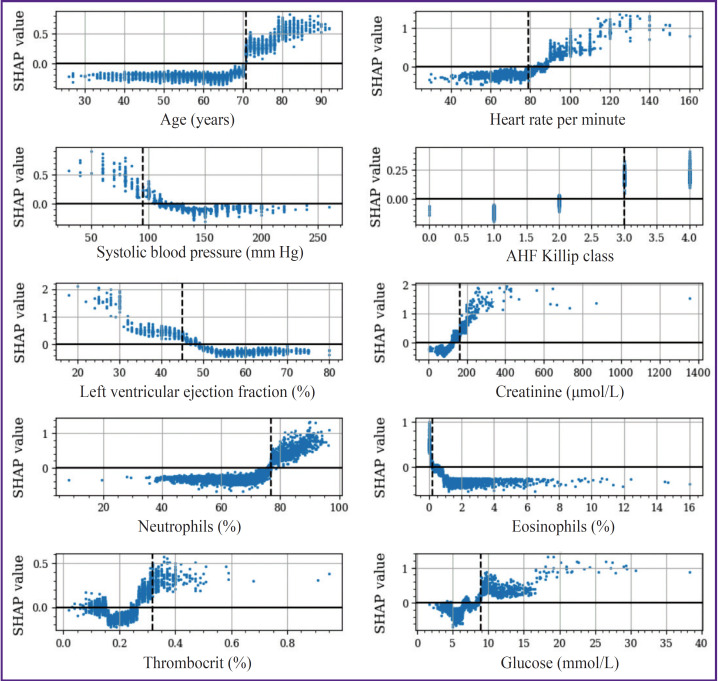
The effect of continuous indicators and their threshold values on the endpoint evaluated by the SHAP method A dashed line designates the cut-off threshold

The results of the study have shown that the threshold values obtained by various methods often differ from each other. For example, the cut-off threshold for LV EF indicator according to SHAP was <45%, while after OR and AUC maximization, the cut-off point was fixed at the level of 31 and 51%, respectively (Table 3). At the same time, the threshold values determined by the SHAP algorithms were closest to the criterial boundaries verified by the Min(p) method. The threshold values obtained by the Max(OR) had an extreme cut-off threshold and allowed for identification of only a narrow group of individuals with a high IHM probability. It is worth mentioning that the SHAP method enables investigators not only to determine the threshold boundaries but to assess the intensity of the impact on the IHM indicators, whose values are in the “risk zone”. The following features are referred to the categorical factors selected by this method: age >70 years, HR >89 per minute, SBP <95 mm Hg, AHF Killip class >II, creatinine >166 μmol/L, LV EF <45%, NEUT >77%, EOS <0.2%, PCT >0.32%, glucose >8.9 mmol/L (Figure 2). With the use of a LV EF diagram as an example, it is seen that the IHM probability increases successively in the value range of 44–31% and grows sharply at the level of this indicator <30%. The elevation of glucose in the blood by more than 8.9 mmol/L increases the risk of IHM, but the probability of the fatal outcome grows significantly at its level exceeding 17 mmol/L.

**Table 3:** Categorization of continuous predictors of in-hospital mortality using various methods

Predictor	Method	Risk factor	p	OR (95% CI)	AUC
Age	Max(OR)	46+	<0.00001	10.7 (3.4–33.6)	0.542
	Min(p)	70+	<0.00001	4.11 (3.26–5.19)	0.655
	Max(AUC)	65+	<0.00001	3.73 (2.92–4.77)	0.658
	Centroid	66.5+	<0.00001	3.65 (2.87–4.63)	0.650
	SHAP	71+	<0.00001	4.11 (3.26–5.19)	0.655
SBP	Max(OR)	60–	<0.00001	31.5 (10.9–91.4)	0.523
	Min(p)	92–	<0.00001	11.0 (8.3–14.7)	0.613
	Max(AUC)	112–	<0.00001	5.3 (4.2–6.8)	0.685
	Centroid	120–	<0.00001	4.8 (3.8–6.0)	0.676
	SHAP	95–	<0.00001	11.6 (8.7–15.4)	0.638
HR	Max(OR)	150+	<0.00001	41.60 (4.32–401.16)	0.506
	Min(p)	95+	<0.00001	6.17 (4.82–7.89)	0.650
	Max(AUC)	82+	<0.00001	4.54 (3.60–5.73)	0.669
	Centroid	79+	<0.00001	3.96 (3.13–5.02)	0.665
	SHAP	89+	<0.00001	5.5 (4.3–7.0)	0.663
AHF Killip class	Max(OR)	4	<0.00001	9.60 (7.41–12.40)	0.662
	Min(p)	4	<0.00001	9.60 (7.41–12.40)	0.662
	Max(AUC)	3+	<0.00001	7.08 (5.59–8.99)	0.711
	Centroid	3+	<0.00001	7.08 (5.59–8.99)	0.711
	SHAP	3+	<0.00001	7.08 (5.59–8.99)	0.711
Creatinine (μmol/L)	Max(OR)	427.0+	<0.00001	30.5 (10.2–91.7)	0.518
	Min(p)	188.6+	<0.00001	13.1 (9.4–18.0)	0.625
	Max(AUC)	122.9+	<0.00001	5.8 (4.5–7.4)	0.701
	Centroid	113.3+	<0.00001	4.6 (3.6–6.0)	0.693
	SHAP	166+	<0.00001	10.0 (7.5–13.4)	0.645
NEUT (%)	Max(OR)	94.2+	<0.00001	23.0 (4.6–114.8)	0.513
	Min(p)	78.8+	<0.00001	9.1 (6.6–12.5)	0.730
	Max(AUC)	75+	<0.00001	11.3 (7.8–16.2)	0.774
	Centroid	74.0+	<0.00001	9.9 (6.9–14.4)	0.751
	SHAP	77+	<0.00001	11.3 (7.8–16.2)	0.774
EOS (%)	Max(OR)	1.3–	<0.00001	9.8 (5.3–18.1)	0.666
	Min(p)	0.3–	<0.00001	7.9 (5.6–11.0)	0.741
	Max(AUC)	0.3–	<0.00001	7.9 (5.6–11.0)	0.741
	Centroid	0.5–	<0.00001	7.6 (5.2–11.1)	0.722
	SHAP	0.2–	<0.00001	6.9 (5.0–9.4)	0.712
LV EF (%)	Max(OR)	31.0–	<0.00001	19.7 (12.1–32.1)	0.594
	Min(p)	31.0–	<0.00001	19.7 (12.1–32.1)	0.594
	Max(AUC)	51–	<0.00001	4.9 (3.5–6.7)	0.690
	Centroid	51–	<0.00001	4.9 (3.5–6.7)	0.690
	SHAP	45–	<0.00001	5.9 (4.3–8.1)	0.648
PCT (%)	Max(OR)	0.36+	<0.00001	4.5 (2.7–7.4)	0.524
	Min(p)	0.36+	<0.00001	4.5 (2.7–7.4)	0.524
	Max(AUC)	0.22+	0.00002	1.8 (1.4–2.4)	0.598
	Centroid	0.21+	0.0009	1.6 (1.2–2.1)	0.576
	SHAP	0.32+	0.00002	1.8 (1.4–2.4)	0.598
Glucose (mmol/L)	Max(OR)	31+	0.002	44.8 (4.0–496.5)	0.505
	Min(p)	8.4+	<0.00001	5.2 (3.9–7.1)	0.620
	Max(AUC)	6.5+	<0.00001	4.9 (3.6–6.8)	0.689
	Centroid	6.9+	<0.00001	5.0 (3.6–6.7)	0.688
	SHAP	8.9+	<0.00001	4.7 (3.5–6.4)	0.631

***At the fourth stage of the study***, MLR-based prognostic IHM models with predictors in a categorical form have been developed (Table 4). The comparative analysis has shown that the majority of models possessed a high predictive capacity irrespective of the method of threshold value determination. The model, in which predictor categorization was done by the Max(OR), was an exception because it did not provide the acceptable prediction accuracy (AUC — 0.662). The evaluation of the model quality metrics with categorical and continuous predictors has demonstrated the absence of statistically significant differences. For example, 95% CI for AUC medians in the analyzed models was 0.882–0.887 and 0.841–0.959, respectively, at p=0.172, which indicated their comparable prognostic accuracy.

**Table 4:** Accuracy assessment of the models for predicting in-hospital mortality based on predictors in the categorical form, Me [Q1; Q3]

Metrics	SHAP	Min(p)	Max(AUC)	Max(OR)	Centroids
AUC	0.885 [0.882; 0.887]	0.876 [0.873; 0.879]	0.896 [0.893; 0.898]	0.662 [0.658; 0.666]	0.888 [0.885; 0.890]
Sen	0.815 [0.809; 0.821]	0.815 [0.809; 0.821]	0.815 [0.809; 0.821]	0.296 [0.289; 0.304]	0.815 [0.808; 0.821]
Spec	0.825 [0.823; 0.826]	0.826 [0.824; 0.828]	0.821 [0.819; 0.822]	0.962 [0.961; 0.962]	0.823 [0.822; 0.825]
PPV	0.149 [0.148; 0.150]	0.147 [0.116; 0.178]	0.148 [0.147; 0.149]	0.240 [0.235; 0.245]	0.150 [0.149; 0.151]
NPV	0.991 [0.991; 0.992]	0.991 [0.991; 0.992]	0.991 [0.991; 0.992]	0.973 [0.973; 0.973]	0.991 [0.991; 0.992]
F1-score	0.250 [0.248; 0.252]	0.251 [0.250; 0.253]	0.251 [0.250; 0.253]	0.271 [0.265; 0.277]	0.254 [0.252; 0.256]

**Figure 3.**
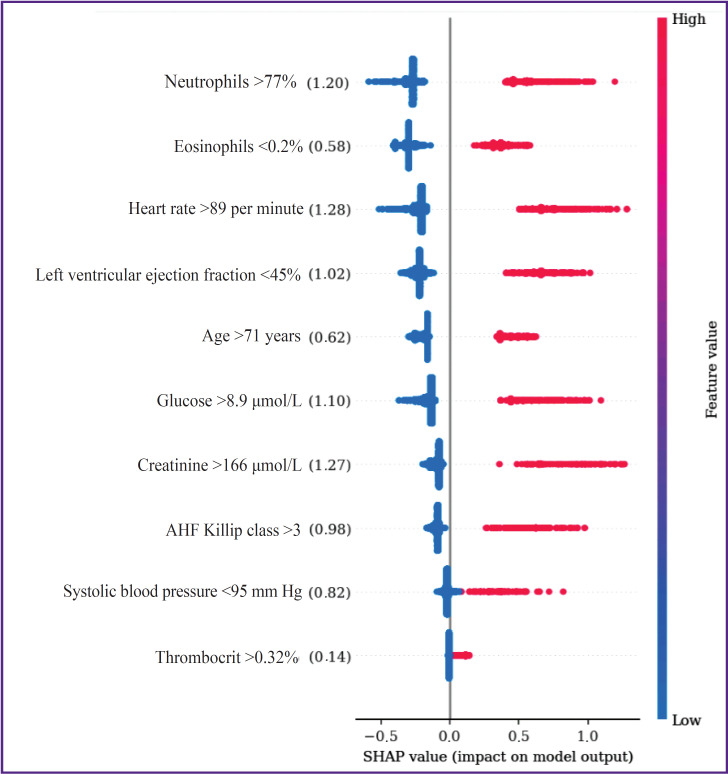
Assessment of importance of in-hospital mortality predictors in the categorical form in the multivariate logistic regression model

The analysis of categorical variable impact on IHM by the SHAP method has shown that the greatest effect on IHM was caused by the following risk factors: HR >89 per minute, creatinine >166 μmol/L, and neutrophil content >77% (SHAP values of 1.28; 1.27, and 1.20, respectively) (Figure 3). Weaker association with the endpoint was observed in the indicators of glucose >8.9 mmol/L, LV EF <45%, AHF (Killip class III and IV), SBP <95 mm Hg (SHAP — 1.10, 1.02, 0.98, 0.82, respectively), while the weakest impact on IHM was produced by the age of >71 years, eosinophil content <0.2%, and thrombocrit >0.32% (SHAP — 0.62, 0.58, and 0.14).

## Discussion

In recent years, machine learning-based prognostic models are being developed, the structure of which is presented by the factors with a higher predictive potential than in the classic scales of riscometry. Perspective tools for their selection are the algorithms of explainable artificial intelligence (XAI), the elements of which include determination of the threshold values of the analyzed factors and assessment of their impact on the endpoint of the investigation. The XAI concept is based on the grounding and interpretation of various decisions obtained as a result of modeling, evaluation of their significance, and accuracy of the generated conclusions [24]. One of the barriers for implementation of these principles is multifactor and nonlinear nature of prognostic models, when a battery of diverse data associated with different causes of fatal events influence the endpoint. Non-transparency of interconnections of various factors with IHM may be partially overcome by their categorization, which allows one to detail the correlations of indicators of clinical and functional status of STEMI patients with the resulting variable. According to the literature data, the most available method of categorization is descriptive statistics with calculation of medians, quartiles, and quantiles [16, 25, 26]. At the same time, a large portion of critical remarks on categorization is connected exactly with this approach, which is caused primarily by the dependence of these threshold values on the specific sample, absence of interconnection with the clinical context, ignoring possible non-linear relations, and others. Another approach is based on the selection of the threshold value, known from practice as going beyond the norm [26]. An alternative method is searching for the optimal threshold values based on minimization or maximization of the target functions. In our opinion, categorization must be considered only within the framework of solving a specific clinical task, although the selected predictors may be of interest for realization of other prognostic investigations. Whatever the method was used to determine their threshold values, they may result in the loss of information, on the one hand, while bringing new knowledge, on the other. In the present study, the threshold values were determined using the methods of grid search to find the optimal cut-off points, calculation of centroids, and SHAP. Deviations from the threshold values increased their predictive potential and allowed us to refer these indicators to the IHM risk factors in STEMI patients after PCI. It has been established that SHAP method, which is considered as a XAI technology, is a promising tool of categorization due to a precise estimation of the cut-off thresholds and analysis of interrelations of predictors in the continuous and categorical forms with the investigation endpoint. Despite the comparable accuracy of the prognostic IHM models with continuous and categorical predictors, there were certain differences in the intensity of their influence on the endpoint. Thus, indicators of LV EF and creatinine demonstrated the greatest interconnection with fatal outcome among the continuous predictors, while the smallest one was shown by AHF Killip class and patients’ age. Among the categorical predictors, the most noticeable association of IHM was with HR >89 per minute, creatinine >166 μmol/L, and neutrophil level >77%, and the minimal association was with thrombocrit >32% and eosinophil content <0.2%. These differences may be explained by the endpoint of the investigation presented in the form of IHM from all causes, which does not allow for verification of the predictors connected with the concrete variant of unfavorable outcome (repeated myocardial infarction, fatal arrhythmias, bleeding, and others). The other cause of mismatched predictor importance may be connected with the fact that in our study, they were categorized with the selection of only one cut-off threshold. A single criterial boundary limits the possibilities for estimation of non-linear interconnections of IHM with the predictor values being in the risk zone. A sharp increase of IHM probability in LV EF less than 30% relative to the range of its values of 31–44% may serve as a convincing example of such situation (see Figure 2). In our case, the LV EF predictor <45% was not so important as indicators HR >89 per minute and creatinine >166 μmol/L in the prognostic IHM model with the categorical factors; besides, this predictor indicated that overcoming criterial boundary, selected in the process of dichotomization, is associated with the growing risk of an adverse outcome. In the recent years, in order to improve the accuracy of prediction, it is recommended to perform variable categorization using several cut-off thresholds, which specifies non-linear interconnections of predictors with the endpoint [19]. The results of our study demonstrate that despite some problems connected with dichotomization of continuous variables in prognostic models, it is appropriate to perform this procedure, since it broadens the possibilities for explanation and clinical interpretation of the generated conclusions. At the same time, it is quite evident that this approach needs further improvement by applying the technologies of multilevel categorization.

**Study limitations** are linked to its retrospective character, application of only dichotomization for categorization of the continuous variables, and model validation using the data from other medical settings.

## Conclusion

In the present investigation, we selected and validated new predictors of in-hospital mortality in patients with ST-segment elevation myocardial infarction after percutaneous coronary intervention, categorized them, and developed prognostic models with continuous and categorical variables based on multivariate logistic regression, random forest, and stochastic gradient boosting. These models had a high and comparable predictive accuracy, but their predictors had different degree of impact on the endpoint. The comparative analysis has shown that Shapley additive explanation method has advantages in categorization of predictors and allows for detailing the structure of their interconnections with in-hospital mortality. To improve prognostic models with categorical predictors, it is appropriate to use multilevel cut-off thresholds upgrading the quality and explanation of the generated conclusions.
